# Detailed Histologic Evaluation of Eosinophilic Esophagitis in Pediatric Patients Presenting with Dysphagia or Abdominal Pain and Comparison of the Histology between the Two Groups

**DOI:** 10.1155/2017/3709254

**Published:** 2017-12-17

**Authors:** Thirumazhisai S. Gunasekaran, Christopher Chu, Nemencio Ronquillo, Rohini Chennuri, Brian Adley, Kristina Borgen, Alan Schwartz, Robert Newberry, James Berman

**Affiliations:** ^1^Advocate Children's Hospital, Loyola Medical Center and University of Illinois, 1775 Dempster Street, Park Ridge, IL 60068, USA; ^2^University of Illinois, Chicago, IL, USA; ^3^Advocate Children's Hospital, 1775 Dempster Street, Park Ridge, IL 60068, USA; ^4^Rady Children's Hospital, San Diego, CA, USA

## Abstract

EoE in children presents with four main symptoms. Most common symptoms exhibited by our clinic population are dysphagia (D) and abdominal pain (AP). Despite similar treatments, we found in an earlier study that the outcomes between these two groups were different. Therefore, we investigated if there exist any histological differences between these groups that could further our knowledge of EoE.* Aim*. To compare esophageal histology in detail, apart from the eosinophil count, between EoE-D and EoE-AP.* Method*. Biopsies of patients with EoE-D and EoE-AP were reevaluated for 10 additional histological criteria, in addition to the eosinophil count.* Results*. Both groups had 67 patients; peak mean eosinophil was 33.9 and 31.55 for EoE-D and EoE-AP (*p* < 0.05). Eosinophilic microabscesses, superficial layering of eosinophils, and epithelial desquamation were twice as common and significant in EoE-D group than EoE-AP. Eosinophil distribution around rete pegs was also significantly higher in EoE-D group. The remaining criteria were numerically higher in EoE-D, but not significant, with the exception of rete peg elongation.* Conclusion*. EoE-D patients have significantly higher eosinophils compared to EoE-AP, and the level of inflammation as seen from eosinophil microabscesses, superficial layering, desquamation, and the distribution around rete pegs is significantly higher.

## 1. Introduction

Based on the Consensus guidelines [1, 2], it is our practice that patients seen at the Eosinophilic Esophagitis Clinic, Advocate Children's Hospital, Park Ridge, IL, are subgrouped, depending on the dominant presenting symptom as EoE–dysphagia (EoE-D), EoE–abdominal pain (EoE-AP), EoE–GERD/vomiting, and EoE–failure to thrive/feeding difficulty (EoE–FTT). In a previous study [3], we showed that the two most common subtypes of EoE seen in our EoE Clinic are EoE-D and EoE-AP and these two groups had different clinical, endoscopic, and histopathologic features. The most striking feature was that, with standard treatments, the two groups had contrasting outcomes. EoE- D had a favorable outcome compared to EoE- AP. Most of the EoE studies in adult patients, where dysphagia is the main symptom, have a favorable outcome with treatment. Based on these variable outcomes, in our previous study, we compared the clinical features of EoE-AP to patients with functional abdominal pain (FAP) with discriminant score and cluster analysis. The result showed that the clinical features were similar, between functional abdominal pain and EoE-AP groups, and only differed in the presence of eosinophils in the esophagus [3]. Since there is no biomarker for EoE, we concluded from this study [3] that “…if optimal diet or pharmacotherapy does not lead to symptomatic improvement or when there is a dissociation between histological and symptomatic improvement, it is worth relooking not only at adherence to diet and/or medications but also managing the EoE-AP group with methods successful in FAP patients.”

While there remains ongoing research to seek a reliable biomarker for the diagnosis of EoE, we sought to see if there are additional histological evidence to confirm the diagnosis and/or to differentiate EoE from esophageal eosinophilia. Consensus Statement recommends [2] that, apart from the eosinophil count, additional histological features and immunohistologic stains can be obtained for a more accurate diagnosis [2]. Based on this recommendation, further studies have included additional histological criteria like eosinophilic abscesses, degranulation, and basal zone hyperplasia or biomarker staining for eosinophil degranulation products like major basic protein and interleukins. But none of these features clinch the diagnosis of EoE. In addition, the diverse presentation of symptoms in pediatric patients adds further challenges for an accurate diagnosis of EoE. Adult patients with EoE have dysphagia as the primary presenting symptom, while in pediatrics it is variable: feeding difficulty, abdominal pain, GERD-like symptoms, and dysphagia [3]. Moreover children presenting with abdominal pain to a clinic, in general, have FAP compared to those presenting with dysphagia having EoE. Based on these differences in presentation and our previous study [3] raising doubt if EoE patients presenting with abdominal pain without dysphagia are truly EoE or are part of a heterogeneous group of diseases with EoE as one of the diagnosis, we raised the issue that further studies are required. As a follow-up on our previous study [3], we sought to look for additional histological criteria within the same group of patients to see if this will give us a better understanding on the pathogenesis of EoE and or diagnostic histological features between EoE-D and EoE-AP patients.

In our previous study [3], apart from the number of eosinophils in the biopsies, we looked at four histologic features retrospectively: eosinophilic microabscesses, basal epithelial hyperplasia, papillomatosis, and spongiosis within the two groups. Eosinophilic microabscesses were significantly more frequent in the EoE- D (*p* < 0.001), while the remaining histologic features were not statistically different between the two groups. In the current study, within the same group of patients, we relooked at an in-depth and detailed reexamination of the histopathology of these two groups of EoE patients, for additional evidence to support our hypothesis.

## 2. Aim

Our hypothesis is that there are differences in the histology of the esophagus between EoE-D and EoE-AP patients. The aim is to compare the diagnostic esophageal histology in detail, of patients with EoE-D and EoE-AP, to see if they are similar or different and if they are different, whether these differences contribute to the understanding of the pathogenesis of the two groups.

## 3. Method

### 3.1. Inclusion and Exclusion Criteria

In this retrospective study all pediatric patients seen at the Eosinophilic Esophagitis Clinic, Advocate Children's Hospital, Park Ridge, IL, over 2 and 1/2 years (1/2010–6/2012) with eosinophilic esophagitis were included in the study. The diagnosis of EoE was made by the following criteria: symptom(s) of esophageal dysfunction as mentioned above and esophageal biopsy showing 15 or more eosinophils per high power field (HPF) on ×400 light microscopy. These patients were pretreated with proton pump inhibitors (PPIs) or had a negative esophageal pH study and had no increased infiltration of eosinophils in the antral or duodenal biopsies [1, 2]. From the four subgroups of EoE patients, EoE-D and EoE-AP patients (based on the dominant presenting symptom) were chosen for comparison, as these were the larger groups. Patients with abdominal pain had central or diffuse abdominal pain and no dysphagia. Patients with celiac disease, Crohn's disease, or achalasia were excluded.

### 3.2. Patient Population and Features

We queried a previously created secure Access (Microsoft, Redmond, WA) database to the following data points: symptoms, physical findings, complete blood count, serum electrolytes, urea, creatinine, liver function profile, sedimentation rate, urinalysis, and endoscopic findings (furrows, white spots/exudates, concentric rings, and friability/crepe paper appearance, entered as absent, 0, or present, 1). The histology of the duodenum, stomach, distal, and mid esophagus were captured. Each patient had two to three biopsies from the descending duodenum, antrum, distal esophagus (2-3 cm above Z-line), and mid esophagus. These features, except detailed histologic evaluation of the degree/stage of eosinophil-rich inflammation, were published in our previous study [3]. The demographics and presenting symptoms of the EoE-D and EoE-AP patients are given in Table 1 and visual EGD findings are in Table 2. The Institutional Review Board, Advocate Children's Hospital, Park Ridge, IL, approved this study.

### 3.3. Histopathologic Analysis

All biopsy specimens were fixed in formalin and stained with hematoxylin and eosin (H&E). H&E stained slides from the two EoE subgroups that met the inclusion criteria were retrieved and reviewed. The initial biopsies leading up to the diagnosis of EoE were considered for extensive review. A careful review of all biopsies and fields was done and the area which had the most dense eosinophilic inflammation, at HPF on ×400 light microscopy, was taken for analysis. Five pathologists through review of current literature and standard pathology texts [1, 2] concluded to analyze ten histological criteria, in addition to the eosinophil count. These criteria evaluated the degree/stage of eosinophil-rich inflammation and are listed in Table 3.

Esophageal biopsies from distal and mid sections of the esophagus were scanned at ×100 power. Intraepithelial eosinophils were quantified at ×400 from the area with maximum eosinophilic density. Only intact eosinophils were considered in determining the peak eosinophil count. The slide with the highest peak eosinophil count was then further evaluated in detail for the ten criteria described earlier.

### 3.4. Statistical Analysis

Data were entered into a secure Microsoft Access Database and statistical analysis was done using SPSS version 20. A *p* value < 0.05 was accepted as statistically significant.

## 4. Results

### 4.1. Patients and Design of Histology Evaluation

During the study period of 2 and 1/2 years we had a total of 73 patients in the two groups and from these 67 patients from each group were selected for further analysis, who fulfilled the diagnostic criteria of EoE and had evaluable biopsies. Six patients who did not fulfill these criteria were excluded. Within the current study groups about 60 patients in each group were part of the previous study [3]. Four pathologists, from the group of five, by reviewing a set of slides, obtained a 98% agreement rate on all set histologic criteria for sample of normal (10 biopsies) and sample of EoE esophageal biopsies (10 biopsies) prior to histologic evaluation of the patients for the current study. Pathologists were blinded to clinical information and EoE subgroups, to limit bias during the slide review process.

### 4.2. Esophageal Histology

**Figure 1:**
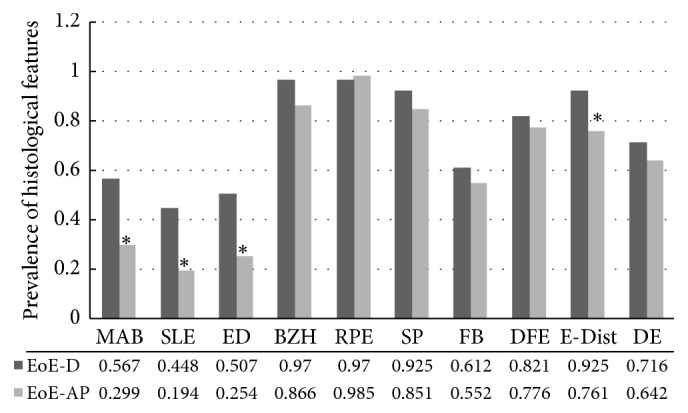
*Comparison of the degree/stage of eosinophil-rich inflammation on the biopsies of EoE-D and EoE-AP patients*. MAB: microabscess, SLE: superficial layering, ED: epithelial desquamation, BZH: basal zone hyperplasia, RPE: rete pegs elongation, SP: spongiosis or increased intracellular space, FB: lamina propria fibrosis, DFE: diffuse or focal distribution, E-Dist: distribution around rete pegs, and DE: degranulation. Fibrosis (FB) was evaluable in 57 (EoE-D) and 54 (EoE-AP) patients and was positive in 41 (71.9%) and 37 (68.5%), respectively, *p* value 0.85 (Chi-square test on 1 df = 0.03, *p* = 0.85). *∗* Statistically significant.

The eosinophil counts for both groups are given in Table 4. EoE-D group had higher eosinophils than EoE-AP and was significant and in both groups the distal biopsy had higher eosinophils than the midesophagus. The detailed histological findings for each of the two groups are shown in Figure 1. Three findings, in particular eosinophilic microabscesses (MAB), superficial layering of eosinophils (SLE), and epithelial desquamation (DE), are noteworthy. These three findings were about two times more common in EoE-D patients than EoE-AP patients and statistically significant. Pattern of eosinophil distribution around the rete pegs also was significantly higher in the EoE-D group. The remaining findings except rete peg elongation were numerically greater in EoE-D patients than EoE-AP, but not statistically significant. In regard to subepithelial/lamina propria fibrosis (FB), this finding was only evaluable if patient samples contained lamina propria in their biopsy and 57 (85%) in EoE-D and 54 (80%) in EoE- AP were evaluable and fibrosis was observed numerically more in EoE-D but was not significant.

## 5. Discussion

Diagnosis of EoE is based on the presence of 15 or more eosinophils [2, 3], the “hallmark” of histological diagnosis, but an exact number required for a definitive diagnosis of EoE continues to be a moving target. As an alternative, some studies support a different method: 20–24 eosinophils on a single biopsy or 15 or more eosinophils on biopsies from two levels for the diagnosis [4, 5]. Since a definitive number of eosinophils, either to make an accurate diagnosis of EoE and/or to exclude other causes of eosinophilic esophageal infiltration, including GERD, is not clear, histological diagnosis based on the number of eosinophils continues to be in dispute. Based on a study on the increasing incidence of EoE, a recent editorial titled “Eosinophilic Esophagitis-Emerging Epidemic or Misdiagnosed Malady?” questions the accuracy of the diagnosis of EoE, for similar reasons [6, 7]. Hence there is a prevailing concern whether clinicians are accurately diagnosing EoE or are lured and misled by the “15 eosinophils” and fail to correlate the eosinophils with symptom(s) of esophageal dysfunction. In addition 2011 Consensus Statement recommends that, apart from the eosinophil count, additional histological features, like the ones described in this study, and immunohistologic stains be obtained for more accurate diagnosis [2]. Therefore the focus shifts to whether additional inflammatory histological criteria would increase the accuracy of the diagnosis of EoE and differentiate and/or eliminate other causes of esophageal eosinophilic inflammation. Another challenge for the clinician is to associate the presenting symptom(s) of esophageal dysfunction, the second criterion, to authenticate the diagnosis of EoE. When patients present with dysphagia, a symptom of esophageal dysfunction, with or without regurgitation/heartburn, the challenge is to differentiate GERD from EoE. When the presenting symptom is abdominal pain without dysphagia, it is more challenging because dysphagia is a cardinal symptom of esophageal dysfunction, whereas abdominal pain does not hold a similar position [8, 9]. This makes the accurate diagnosis of EoE in the EoE-AP group even more difficult, as there are multiple causes for central abdominal pain. So it raises the question: in the absence of esophageal dysfunction, are the histological findings in EoE-AP consistent with a diagnosis of EoE?

Though our study evaluated the eosinophilic inflammation at one point, to better understand the differentiating histological features of EoE, we reviewed “the evolution” of the histological changes in EoE. With antigen insult to the esophagus, eosinophilic inflammation begins in the peripapillary area, an area closest to the vasculature. Superficial and diffuse distribution represents later stage of epithelial infiltration. Basal zone hyperplasia and lengthening of lamina propria papillae are secondary changes to antigen insult and may be severe with increased duration of the insult [10]. Another process contributing to inflammation is degranulation of eosinophils which correlates with intraepithelial eosinophilia or degree of mucosal inflammation. Degranulation leads to cytotoxin and cytokine release, resulting in the desquamation or degeneration of cells, and mobilization of more eosinophils. Odze classified the histological findings of EoE as major: increased eosinophils (greater than 15), eosinophilic microabscesses, superficial layering of the eosinophils, surface sloughing of squamous cells, and degranulation of eosinophils, and minor: “marked” basal cell hyperplasia, lengthening of the lamina propria papillae, increased intraepithelial lymphocytes and mast cells, increased intracellular edema, and increased lamina propria fibrosis [10].

Odze's classification of EoE histology and other studies [4, 5, 10] support the fact that, apart from the eosinophil count, additional features, eosinophilic microabscesses, superficial layering, desquamation, and degranulation are predominantly seen in EoE compared to GERD. Hence these criteria are considered additional features to diagnose EoE and to exclude GERD, another disease which manifests with esophageal eosinophilic inflammation. Collins et al. have recently shown that these additional histological criteria are useful in the diagnosis and monitoring of EoE patients [11]. The three features, eosinophilic microabscesses, superficial layering, and desquamation, which our study showed to be significant, are recurring findings and are seen significantly more in EoE and have been used to differentiate it from GERD [12–19]. Our study came to the same conclusion and validates that EoE-D is clearly EoE with these features. On the contrary since these histological features are not significantly seen in EoE-AP, it suggests that EoE-AP may be more of a heterogeneous group of diseases and that should be the subject of future studies.

Does above difference in the inflammation in EoE-D and EoE-AP help understand the pathogenesis of the primary symptom(s), dysphagia and central abdominal pain, of the EoE groups? Our study showed that the epithelium of the esophagus in EoE-D has more histological inflammation in comparison to EoE-AP, which is also supported by the increased endoscopic findings of exudates and furrows in the EoE-D group. This epithelial inflammation, as well as release of cytokines or chemokines from the degranulation of eosinophils, results in increased smooth muscle reactivity and dysmotility of the esophagus resulting in dysphagia, as seen in adults with inflammatory type of EoE with dysphagia [20–22]. Eosinophilic inflammation in the duodenum was shown to be associated with abdominal pain via the release of cytokines [21, 22], but it is not clear if inflammation in a proximal organ, the esophagus, can lead to abdominal pain at a distal site. Lamina propria fibrosis, seen in about 80% of the biopsies, was not significantly different in the two groups. We were expecting that the fibrosis would be significantly more in EoE-D and we were surprised that the results did not support it. Whether the results would change if all biopsies were evaluable for fibrosis is left to speculation.

Ours is the first to systematically compare 10 additional histological criteria, apart from the eosinophil count, in EoE-D and EoE-AP at diagnosis. Limitations of our study are as follows. Although the biopsies were reviewed prospectively, patient data is retrospective and did not correlate the eosinophil counts with the rest of the inflammatory findings. Not all biopsies were evaluable for subepithelial fibrosis and they did not include immunohistological staining. These are opportunities for future studies.

In conclusion, and in accordance with Consensus and Collins' recommendations [1, 2, 11], pathologists should describe additional above listed inflammatory features, in addition to the eosinophil count, when providing histology reports on patients with EoE. Four features, eosinophil microabscesses, superficial layering, epithelial desquamation, and distribution of eosinophils around rete pegs, are significantly and consistently seen more often in EoE-D than in EoE-AP patients. While these additional features explain pathogenesis of dysphagia in patients with EoE-D, their absence in EoE-AP suggests that EoE-AP may be a more heterogeneous group of diseases including EoE, GERD, and other causes of esophageal eosinophilia.

## Figures and Tables

**Table 1:** Demographics and Symptoms of Patients with Eosinophilic Esophagitis.

	EoE-D *n* = 67 (%)	EoE-AP *n* = 67 (%)	*p* value
Male	59 (88.1)	52 (77.6)	0.11
Mean age, yr (range)	11.8 (3–17)	9.45 (2–17)	0.001
Presenting symptoms^a^			
Dysphagia	67 (100)	1^b^ (0.01)	<0.001
Abdominal pain	8 (11.9)	66 (98.5)	<0.001
Nausea	11 (16.4)	31 (46.2)	<0.001
Vomiting	12 (17.9)	11 (16.4)	0.82
Regurgitation	5 (7.5)	7 (10.4)	0.55
Heartburn	7 (10.4)	6 (9.0)	0.77

^a^Some patients had more than one presenting symptom. ^b^Patient initially presented with dysphagia; however, subsequent visits showed abdominal pain as the predominant symptom.

**Table 2:** Endoscopic findings of patients with EoE.

EGD number (%)	EoE-D (*n* = 67)%	EoE-AP (*n* = 67)%	* p *value
Linear furrows	55 (82.1)	34 (50.7)	<0.001
White exudates	34 (50.7)	17 (25.4)	0.003
Linear furrows and white Exudates	32 (47.8)	10 (14.9)	<0.001
Concentric rings	7 (10.4)	3 (4.5)	0.19
Tears/crepe paper appearance	4 (6.0)	0 (0)	0.12

**Table 3:** Histological criteria evaluating the degree/stage of eosinophil-rich inflammation.

(1)	Eosinophilic microabscesses (MAB)	Four or more eosinophils clustered together.

(2)	Superficial layering of eosinophils (SLE)	Superficial infiltrate of eosinophils (>1 eosinophil at ×400).

(3)	Epithelial desquamation (ED)	Degenerative (i.e., necrotic, pyknotic-dense, and dark nuclei because of nuclear shrinkage due to irreversible condensation of chromatin in the nucleus of a cell undergoing necrosis or apoptosis, dyskeratotic-deep pink cytoplasm due to abnormal keratinization occurring prematurely within individual cells, or groups of cells below the stratum granulosum) squamous epithelial cells.

(4)	Basal zone hyperplasia (BZH)	Basal cells occupying more than 20% of total mucosal thickness.

(5)	Rete peg elongation (RPE)	Rete peg elongation that reaches at least 2/3 of total mucosal thickness.

(6)	Spongiosis (SP)	Edema or dilated intercellular spaces between epithelial cells.

(7)	Subepithelial fibrosis or lamina propria fibrosis (FB)	Evaluated if lamina propria was present in the specimen; collagen fibrils are densely packed and individual collagen fibrils cannot be distinguished.

(8)	Degree of involvement of eosinophils (DFE)	Focal or diffuse; focal is defined as when eosinophils are localized to one fragment of the biopsy while diffuse is when eosinophils are found >1 fragment of the biopsy.

(9)	Pattern of distribution of eosinophils if present (E-Dist)	Eosinophils confined to or around rete pegs (peripapillary), diffusely distributed, or superficially distributed.

(10)	Eosinophilic degranulation (DE)	Presence of free eosinophil granules.

**Table 4:** Eosinophil counts on the esophageal biopsies.

	EoE-D mean (sd) *N* = 67	EoE-AP mean (sd) *N* = 67	*p* value
Peak eosinophil count (mean)	33.91 (5.78)	31.55 (4.96)	0.013
Distal esophagus	33.61 (5.85)	31.46 (5.11)	0.025
Mid esophagus	27.04 (5.89)	24.73 (5.89)	0.015
Mean of distal and mid	30.33 (5.36)	28.10 (4.78)	0.012

In both groups the distal biopsy had higher eosinophils than the midesophagus, except two patients in EoE-D and one patient in EoE-AP group where the mid esophagus had a higher esophageal count.
